# Circulating Cell-Free DNA in Psychiatric Disorders: Current Evidence, Inflammation-Based Stratification, and Future Perspectives

**DOI:** 10.3390/ijms27125285

**Published:** 2026-06-11

**Authors:** Chiara Galbiati, Erika Vitali, Cristian Bonvicini, Roberta Ghidoni, Annamaria Cattaneo

**Affiliations:** 1 Biological Psychiatry Unit, IRCCS Istituto Centro San Giovanni di Dio Fatebenefratelli, 25125 Brescia, Italy; c.galbiati@fatebenefratelli.eu (C.G.); evitali@fatebenefratelli.eu (E.V.); 2 Molecular Markers Laboratory, IRCCS Istituto Centro San Giovanni di Dio Fatebenefratelli, 25125 Brescia, Italy; cbonvicini@fatebenefratelli.eu (C.B.); rghidoni@fatebenefratelli.eu (R.G.); 3 Department of Pharmacological and Biomolecular Sciences, University of Milan, 20122 Milan, Italy

**Keywords:** psychiatric disorders, cell-free DNAs, mitochondrial cell-free DNAs, inflammation, stress

## Abstract

Psychiatric disorders represent a leading cause of disability worldwide and are characterized by substantial biological and therapeutic heterogeneity. Despite significant research efforts, peripheral biomarkers capable of guiding diagnosis, patient stratification, and personalized treatment selection are still lacking. Circulating cell-free DNA (cfDNA) has recently emerged as a promising candidate biomarker, as it may integrate signals of cellular damage, apoptotic activity, and immune activation across multiple tissues. Beyond its role as a marker, cfDNA may also actively contribute to disease processes by functioning as a damage-associated molecular pattern (DAMP), thereby perpetuating inflammatory signaling. The mitochondrial component of cfDNA (cf-mtDNA), which also possesses strong immunostimulatory properties, represents a particularly sensitive indicator of mitochondrial vulnerability to stress. In this context, the present review aims to synthesize the most recent evidence on cfDNA and cf-mtDNA in major psychiatric disorders, including major depressive disorder (MDD), bipolar disorder (BD), and schizophrenia (SCZ). Specifically, we examine their association with psychological stress exposure and childhood trauma, as well as their involvement in inflammation-related pathophysiological mechanisms such as mitochondrial dysfunction, oxidative stress, and hypothalamic–pituitary–adrenal (HPA) axis dysregulation. Available evidence suggests that alterations in cfDNA may be present in subgroups of patients with MDD, BD, and SCZ. However, findings remain heterogeneous and sometimes contradictory, partly due to methodological limitations, including the lack of standardized analytical protocols and insufficient control for potential confounders. Nevertheless, cfDNA holds promise as a tool for inflammation-based patient stratification and for informing personalized therapeutic strategies. Future research directions include the integration of cfDNA within multi-omics frameworks, the analysis of cfDNA methylation profiles to infer tissue of origin, and the exploration of pharmacological strategies aimed at modulating cfDNA as a potential therapeutic target.

## 1. Introduction

Psychiatric disorders constitute a leading cause of disability worldwide and are characterized by marked clinical, biological, and therapeutic heterogeneity. Despite advances in genetics, neuroimaging, and psychopharmacology, psychiatry still lacks robust peripheral biomarkers capable of guiding diagnosis, patient stratification, and personalized treatment selection [1,2]. This limitation contributes to suboptimal treatment outcomes and high rates of chronicity and comorbidity.

In recent years, increasing attention has been directed toward systemic biological alterations associated with psychiatric illness. Evidence suggests that immune dysregulation [3], systemic inflammation [4], oxidative stress [5], mitochondrial dysfunction [6], and metabolic abnormalities [7] represent central components of disease pathophysiology in subsets of patients. These processes are dynamic, transdiagnostic, and closely linked to environmental stressors, lifestyle factors, and medical comorbidities. Accordingly, there is a growing need for biomarkers capable of integrating information across multiple biological systems and capturing cumulative physiological burden. In this context, approaches based on circulating biomarkers—widely adopted in other fields such as oncology [8]—represent a promising strategy for psychiatric research.

Among circulating biomarkers, circulating cell-free DNA (cfDNA) has emerged as a particularly relevant candidate reflecting fundamental biological processes such as cellular damage, apoptotic activity, and immune activation [9]. Unlike circulating tumor DNA, which originates from malignant cells, cfDNA is released from multiple tissues under both physiological and pathological conditions, making it particularly relevant for psychiatric research. cfDNA consists of short DNA fragments, typically around 160–180 base pairs in length, that are released into the bloodstream primarily through apoptosis, necrosis, neutrophil extracellular trap formation, and active secretion via extracellular vesicles [10,11,12]. Moreover, its short half-life enables cfDNA levels to dynamically reflect ongoing biological processes.

Importantly, cfDNA is not merely a downstream marker of cellular damage but may actively contribute to pathological processes by functioning as a damage-associated molecular pattern (DAMP) capable of perpetuating inflammatory signaling and other pathophysiological responses [13,14]. Under conditions of stress or tissue damage, cfDNA has been shown to bind to immune receptors such as Toll-like receptors (TLRs) and cytosolic inflammasome sensors, thereby potentially triggering signaling cascades that promote both local and systemic inflammation [15]. Therefore, cfDNA may function neither exclusively as a passive cellular product nor solely as an active driver of inflammatory signaling, but rather function as both. The biological effects of cfDNA also depend on specific molecular characteristics, including GC content, oxidation state, and concentration, which may differentially activate pro-inflammatory pathways mediated by the transcription factor NF-κB, cytoprotective mechanisms such as NRF2, or apoptosis and hormetic survival responses [16,17]. Taken together, these observations suggest that the potential dual role, as both a marker and mediator of inflammation, could place cfDNA at the intersection of cellular damage and immune activation—two processes increasingly implicated in psychiatric disorders.

cfDNA can be quantified and characterized using several analytical approaches, including quantitative PCR, digital droplet PCR, fluorometric assays, and Next-Generation Sequencing. Emerging metrics include cfDNA fragmentation profiles, integrity indices, and epigenetic signatures such as methylation patterns that may allow inference of tissue of origin. However, cfDNA measurements are highly sensitive to both pre-analytical and analytical variables. Sample type (plasma versus serum), processing time, physical activity, acute stress, smoking status, body mass index, infections, and pharmacological treatments can all influence cfDNA levels [18,19]. These factors represent major potential confounders which can be particularly prevalent in psychiatric populations. For instance, many pharmacological treatments, including antipsychotics, mood stabilizers and antidepressants, are associated with weight gain, insulin resistance, dyslipidemia and metabolic diseases [20], all conditions that could independently elevate cfDNA levels [10]. Similarly, medication-induced changes in immune function, mitochondrial activity and oxidative stress may directly alter cfDNA release, independently of the underlying psychiatric condition. These pharmacological and metabolic confounders are often poorly controlled in existing studies and represent major sources of results variability, hence highlighting the importance of standardized protocols and carefully controlled study designs.

An additional layer of biological relevance is provided by the mitochondrial component of circulating cell-free DNA (cf-mtDNA). Unlike nuclear cfDNA, mitochondrial DNA is structurally and functionally distinct due to its bacterial evolutionary origin, absence of protective histones, and high content of unmethylated CpG motifs [21,22]. These features confer strong immunostimulatory properties to cf-mtDNA, rendering it a potential endogenous DAMP capable of activating innate immune receptors such as Toll-like receptor 9 (TLR9) and cytosolic DNA-sensing pathways [23,24].

Both acute and chronic stress can compromise mitochondrial integrity through sustained glucocorticoid exposure and sympathetic nervous system activation, leading to increased production of reactive oxygen species (ROS) and impaired mitophagy, thereby promoting cell death [25]. Studies in animal models further suggest that acute and chronic stress disrupt mitochondrial dynamics through glucocorticoid-mediated activation of glucocorticoid receptor NR3C1 signaling, ultimately contributing to mitochondrial dysfunction and hypothalamic neuronal injury [26]. In this context, cf-mtDNA may serve as a sensitive marker of mitochondrial vulnerability to stress and could represent an early pre-apoptotic signal.

Mitochondrial DNA is considered particularly susceptible to oxidative damage due to its proximity to sites of ROS production and limited DNA repair capacity [27]. Consequently, mitochondrial dysfunction, oxidative stress, and impaired mitophagy, processes increasingly associated with psychiatric disorders, may represent key drivers of mtDNA release into the circulation [6,28]. Importantly, cf-mtDNA may exert disproportionate biological effects relative to its concentration, amplifying inflammatory signaling even at low levels. This could position cf-mtDNA as a biologically relevant mediator at the intersection of cellular stress, mitochondrial dysfunction, and immune activation in psychiatric disorders.

## 2. cfDNA, Psychological Stress, and Childhood Trauma

A growing body of evidence indicates that psychological stress may represent one of the most biologically relevant drivers of cfDNA release [29,30,31]. Both acute and chronic stress exposures have been associated with increased cfDNA concentrations, likely reflecting stress-induced cellular damage, apoptotic processes, and immune activation [30,32,33]. Experimental studies employing acute stress paradigms have consistently demonstrated rapid increases in cfDNA levels following psychological stress exposure [29,30,32]. Similar findings were reported in naturalistic settings, where elevated cfDNA concentrations were observed in students before academic examinations, followed by a decline after the stressor had resolved [34]. Increased cfDNA levels have also been detected in chronically stressed university students [35]. In this cohort, cfDNA concentrations positively correlated with several hematological and metabolic parameters, including cholesterol levels, suggesting a potential link between cfDNA dynamics, stress-related physiological alterations, and metabolic changes [35].

Chronic psychological stress may also influence cfDNA levels through sustained activation of the hypothalamic–pituitary–adrenal (HPA) axis and the sympathetic nervous system (SNS) [36]. These processes promote oxidative stress, mitochondrial dysfunction, and immune dysregulation, which in turn may contribute to DNA damage and its release into circulation [37]. Consistent with this mechanism, Czamanski-Cohen et al. [38] reported lower plasma cfDNA levels in women undergoing in vitro fertilization who received structured psychological stress-reduction interventions compared with those receiving standard care. Taken together, these findings suggest that circulating cfDNA may represent a cumulative biological index reflecting prolonged exposure to psychological stress across multiple physiological systems.

Within this framework, mitochondrial cell-free DNA (cf-mtDNA) appears to play a particularly relevant role in stress-related cfDNA dynamics. Released in response to cellular stress, mitochondrial damage, and cell death, cf-mtDNA may reflect intracellular processes related to oxidative stress, inflammation, and apoptosis [39]. Several studies have reported increased cf-mtDNA levels in individuals exposed to psychological stress, supporting its role as a potential stress-sensitive biomarker [40]. Acute psychological stress challenges have been shown to induce rapid increases in circulating cf-mtDNA levels [30], sometimes independently of nuclear cfDNA changes [41]. However, findings remain complex. In a study comparing individuals exposed to acute stress with control conditions, plasma cf-mtDNA levels increased in both groups alongside elevations in IL-6. Notably, cf-mtDNA exhibited a dynamic temporal pattern characterized by an early increase (5–10 min), followed by a transient decrease and a second larger peak at 45–75 min, with a slightly stronger response under stress conditions [42]. These observations highlight the dynamic and context-dependent regulation of cf-mtDNA release.

Trauma exposure represents another key factor potentially influencing cfDNA biology. Traumatic experiences have been associated with chronic low-grade inflammation, altered HPA-axis regulation, telomere shortening, and epigenetic modifications [37,43], biological processes closely linked to cfDNA release and turnover. Early life adversity and childhood trauma are particularly relevant in this context, as they constitute well-established risk factors for psychiatric disorders and are associated with persistent biological alterations across the lifespan [44,45]. Experiences such as childhood maltreatment, neglect, and exposure to adverse environments have been linked to sustained immune activation, elevated inflammatory tone, dysregulated HPA-axis activity, and accelerated biological aging [46,47], all mechanisms that may contribute to increased cfDNA release [48]. Notably, to date, no studies have examined total cfDNA in relation to trauma, but research exclusively focused on cf-mtDNA.

Some empirical evidence supports this hypothesis. Morrison and colleagues reported significantly elevated plasma cf-mtDNA levels in women with a history of sexual trauma, particularly when the traumatic exposure occurred during adolescence (ages 14–17) [49]. However, the relationship between cf-mtDNA and trauma-related psychiatric disorders remains inconsistent. For example, a study conducted among trauma-exposed male veterans found no overall differences in cf-mtDNA levels between individuals with and without post-traumatic stress disorder (PTSD), although after adjusting for age, diabetes status, and antidepressant treatment, individuals with PTSD exhibited lower cf-mtDNA levels compared with trauma-exposed controls [50]. These mixed findings suggest that cf-mtDNA alterations may depend on factors such as timing of trauma exposure, physiological adaptation, and comorbid conditions.

Overall, although evidence remains limited, cfDNA, particularly its mitochondrial component, may represent a promising biomarker capturing the biological embedding of stress and trauma. By reflecting alterations in cellular integrity, mitochondrial function, and immune activation, cfDNA dynamics may provide insight into the mechanisms linking psychological stress and early-life adversity to long-term mental health outcomes.

## 3. Circulating Cell-Free DNA Across Psychiatric Disorders

The growing interest in cfDNA within psychiatric research reflects a broader conceptual shift toward understanding mental disorders as systemic conditions involving complex interactions between the brain and peripheral biological systems. Accumulating evidence indicates that immune activation, oxidative stress, mitochondrial dysfunction, metabolic alterations, and dysregulated stress responses could not merely be secondary consequences but may represent core components of the pathophysiology of major psychiatric disorders [6,51,52]. However, these biological alterations are not uniformly present across individuals sharing the same diagnosis, highlighting the need for biomarkers capable of capturing this heterogeneity in a dynamic and clinically meaningful manner.

**Figure 1:**
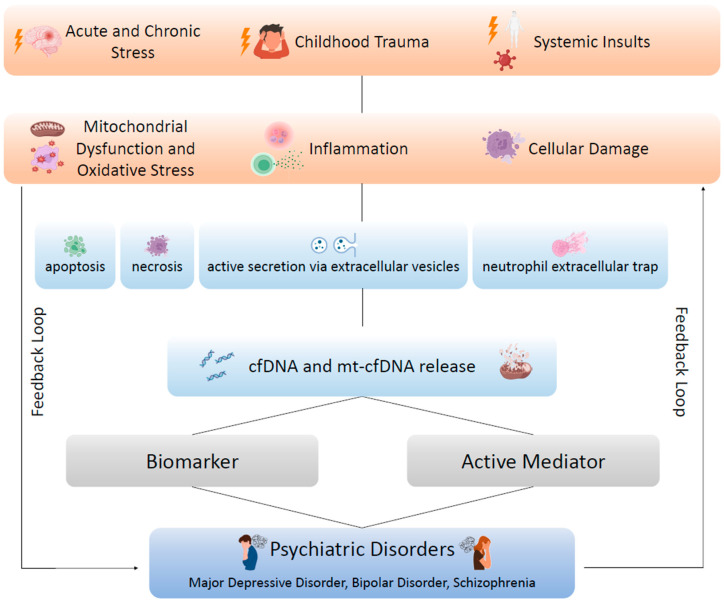
Triggers of cfDNA and cf-mtDNA release and their downstream role in psychiatric disorders.

Within this framework, cfDNA has emerged as a promising candidate biomarker due to its ability to integrate signals of cellular damage, apoptotic activity, and immune activation originating from multiple tissues. Unlike traditional biomarkers that reflect isolated biological pathways, cfDNA has been proposed as a cumulative indicator of systemic stress, encompassing both physical and psychological processes [29,30]. Moreover, the short half-life of circulating cfDNA allows it to rapidly respond to physiological changes, making it particularly suitable for longitudinal monitoring and for capturing state-dependent biological fluctuations [53]. Importantly, cfDNA is not only a passive marker of cellular injury but has also been proposed to actively contribute to immune and inflammatory regulation, thereby linking tissue damage to sustained inflammatory signaling [54,55] (Figure 1).

In recent years, psychiatric research has increasingly explored the potential of cfDNA as a biomarker for several clinical aspects of mental disorders, including diagnosis, prognosis, treatment response, and disease monitoring. Alterations in cfDNA levels and molecular characteristics have been reported across a range of psychiatric conditions, suggesting the involvement of shared biological mechanisms and highlighting their potential as non-invasive biomarkers [56]. Nevertheless, important gaps remain regarding the role of different cfDNA components, such as mitochondrial, genomic, or total cfDNA, across specific psychiatric disorders. For example, a recent meta-analysis reported limited and inconclusive evidence for bipolar disorder (BD) and Major Depressive Disorder (MDD), largely due to the small number of available studies, whereas more consistent findings indicate elevated cfDNA levels in plasma and serum of individuals with Schizophrenia (SCZ) [56,57].

In the following sections, we review and critically synthesize the available evidence on cfDNA in MDD, BD, and SCZ (Table 1), with particular attention to its relationship with psychological stress exposure, inflammatory activation, and underlying pathophysiological mechanisms.

### 3.1. Major Depressive Disorder

MDD is increasingly recognized as a multisystemic disorder characterized by dysregulation across immune, metabolic, neuroendocrine, and mitochondrial pathways. This reconceptualization has stimulated interest in peripheral biomarkers that could reflect the biological burden associated with depressive states. cfDNA could be a promising candidate in this context, as it reflects several processes consistently implicated in MDD, such as cellular damage, apoptotic activity, and immune activation [58,59]. However, a limited number of studies have examined cfDNA in MDD patients, with discordant results—notably, almost all studies conducted in MDD patients to date have exclusively focused on cf-mtDNA.

Some studies have demonstrated lower cf-mtDNA levels in patients with MDD compared with healthy controls [60,61]. Patients with both current and remitted depression showed lower cf-mtDNA levels compared with controls [60], whereas other evidence indicated a positive correlation between cytokine levels (GM-CSF, IL-2 and IL-4) and cf-mtDNA [61]. Conversely, other investigations revealed contrasting results: higher cf-mtDNA levels were found in late-life depression patients [62,63], particularly in those with frailty [63], and in MDD patients [64]. This heterogeneity may partly reflect age-related biological differences, as older individuals are characterized by increased mitochondrial damage and chronic low-grade inflammation (“inflamm-aging”), which may promote cf-mtDNA release [65,66]. Interestingly, cf-mtDNA levels differed between responders to selective serotonin reuptake inhibitors and non-responders, as the former showed lower cf-mtDNA levels after 8 weeks of treatment, while the latter showed no changes [64]. Cf-mtDNA may therefore help delineate biologically distinct subtypes of treatment response. Moreover, adult suicide attempters with various psychiatric diagnoses, among which mood disorders were the most prevalent, showed higher plasma cf-mtDNA levels than healthy controls [37]. Interestingly, a higher cortisol response to the dexamethasone suppression test was found to correlate with these higher levels of cf-mtDNA, suggesting a possible connection between cf-mtDNA levels and hyperactivity of the HPA axis. This observation aligns with the concept of depression as a disorder of allostatic overload, in which sustained stress exposure leads to cumulative cellular damage and impaired physiological resilience. Supporting this notion, depression has been associated with markers of accelerated biological aging, including telomere shortening and mitochondrial dysfunction, both of which may contribute to increased cfDNA release [67,68]. However, other studies found no alterations in cf-mtDNA levels [69] or in cfDNA levels [70] in MDD patients, further complicating the already contradictory results, although an increased levels of C-reactive protein suggested an inflammatory condition [69]. Overall, cf-mtDNA might be relevant in MDD given the extensive evidence linking depression to mitochondrial dysfunction, impaired oxidative phosphorylation, and altered cellular energetics. Depressed individuals show reduced mitochondrial efficiency and increased oxidative stress [71,72], conditions that may favor mtDNA damage and extracellular release.

To date, there is still a lack of literature regarding the role of cfDNA in MDD, and the data published about cf-mtDNA are contradictory. As reported by a meta-analysis conducted in 2023 by Melamud and colleagues, cf-mtDNA levels are not significantly different in MDD compared to healthy subjects [57], underlying the need for further studies to clarify their role in MDD.

### 3.2. Bipolar Disorder

BD is a chronic illness characterized by episodic mood disturbances and progressive functional impairment [73]. Similarly to MDD, elevated inflammatory levels characterize BD and may contribute to cfDNA dysregulation [74]. Specifically, some studies have reported increased levels of cfDNA in BD patients, compared with healthy individuals [75,76], although the limited and contrasting literature support the need for further investigation regarding the role of cfDNA in BD [57,70]. Interestingly, a percentage of cfDNA observed in BD is attributable to the release of cf-mtDNA from mitochondrial dysfunction [77], although to date the literature remains conflicting. Evidence shows both lower [61] and higher [75] cf-mtDNA levels in BD patients and no significant differences between patients and healthy individuals [78,79]. Conversely, higher cf-mtDNA have already been associated with greater brain structural metrics in BD, such as greater patients’ prefrontal cortex surface area and volume [78]. Mitochondrial dysfunction in BD may contribute to the activation of innate immune pathways and to cytokines release [80]. Cf-mtDNA may sustain the vicious cycle linking mood episodes and inflammation and promote neuroprogression, namely the mechanism through which recurrent mood episodes might promote cumulative damage to neural cells and enhance the development of future illness episodes [81]. Elevated oxidative stress associated with repeated manic episodes [82] may trigger cumulative cellular damage, as reflected by persistently elevated cfDNA levels that, in turn, sustain inflammation and trigger the inflammatory signaling [81].

In the context of BD, the disease phase may represent a critical variable, given that BD is characterized be alternating and episodic mood states, which could be associated with distinct cfDNA profiles. Furthermore, cfDNA levels may also be influenced by pharmacological treatments commonly used in BD. For example, a study investigating the influence of drugs on cf-mtDNA observed a reduction in cf-mtDNA levels after one month of treatment with valproate combined with quetiapine and lithium combined with antipsychotics, although no correlation was found between cf-mtDNA and clinical symptoms [75]. Lithium is known to exert neuroprotective [83], anti-inflammatory [84], and mitochondrial-stabilizing effects [85], and this could be associated with its capacity in modulating cfDNA and cf-mtDNA release.

### 3.3. Schizophrenia

SCZ is a multisystem disorder characterized by several cognitive deficits and social dysfunction, alongside immune dysregulation, oxidative stress, endothelial dysfunction, and metabolic abnormalities [86,87]. Consistent evidence has shown an increase in cfDNA levels in SCZ patients [57,70,88,89,90,91], even though some studies have found no differences in cfDNA levels between SCZ and healthy individuals [92], thus highlighting the need for further investigations. Additionally, differences in cfDNA length were observed in SCZ patients, although contrasting results reported both shorter and longer fragments in SCZ patients, compared to healthy individuals [70,90]. cfDNA levels in SCZ patients may be both a consequence and a cause of an inflammatory condition.

**Table 1:** Reports of circulating cfDNA concentrations in Schizophrenia, Bipolar Disorder, and Depressive Disorders. Arrows indicate cfDNA concentration levels in patients compared to healthy controls (↑ increased, ↓ decreased, ↔ no significant difference).

Reference	Year	Diagnosis	Sample	DNA Type	Detection Method	DNA Levels
Depressive Disorder						
[60] Fernström et al.	2021	MDD	Plasma	cf-mtDNA	qRT-PCR	↓
[61] Kageyama et al.	2018	MDD, BD, SCZ	Plasma	cf-mtDNA	qRT-PCR	↓
[62] Gonçalves et al.	2021	Late-life depression	Plasma	cf-mtDNA	qRT-PCR	↑
[63] Ampo et al.	2022	Late-life depression	Plasma	cf-mtDNA	qRT-PCR	↑
[64] Lindqvist et al.	2018	MDD	Plasma	cf-mtDNA	Multiplex RT-PCR	↑
[37] Lindqvist et al.	2016	Suicide attempters with and without MDD	Plasma	cf-mtDNA	qRT-PCR	↑
[69] Behnke et al.	2023	MDD	Serum	cf-mtDNA	Modular RT-PCR	↔
[70] Jiang et al.	2018	MDD, BD, SCZ	Plasma	cfDNA	FCS, Fluorometric quantification, qRT-PCR	↔
Bipolar Disorder						
[75] Teng et al.	2024	BD	Plasma	cf-mtDNA	qRT-PCR	↑
[76] Stertz et al.	2015	BD	Serum	cfDNA	qRT-PCR	↑
[78] Shao et al.	2024	BD	Serum	cf-mtDNA	qRT-PCR	↔
[79] Jeong et al.	2020	BD	Serum	cf-mtDNA	qRT-PCR	↔
[70] Jiang et al.	2018	MDD, BD, SCZ	Plasma	cfDNA	FCS, Fluorometric quantification, qRT-PCR	↔
[61] Kageyama et al.	2018	MDD, BD, SCZ	Plasma	cf-mtDNA	qRT-PCR	↓
Schizophrenia						
[70] Jiang et al.	2018	MDD, BD, SCZ	Plasma	cfDNA	FCS, Fluorometric quantification, qRT-PCR	↑
[88] Ershova et al.	2019	SCZ	Plasma	cfDNA	NQH	↑
[89] Chen et al.	2021	SCZ	Plasma	cfDNA	qRT-PCR	↑
[90] Xue et al.	2025	SCZ	Plasma	cfDNA	cfDNA Sequencing	↑
[91] Li et al.	2024	SCZ	Plasma	cfDNA	FCS, qRT-PCR	↑
[92] Ouyang et al.	2021	SCZ	Plasma	cf-mtDNA	qRT-PCR	↔
[61] Kageyama et al.	2018	MDD, BD, SCZ	Plasma	cf-mtDNA	qRT-PCR	↔

Abbreviations: MDD, Major Depressive Disorder; BD, Bipolar Disorder; SCZ, Schizophrenia; cf-mtDNA, mitochondrial cell-free DNA; cfDNA, circulating cell-free DNA; FCS, Fluorescence Correlation Spectroscopy; NQH, Nonradioactive Quantitative Hybridization.

Evidence from the literature supports the presence of systemic inflammation in SCZ, as indicated by elevated levels of pro-inflammatory cytokines, including TNF-α, IL-8, IL-6, and IL-1β [93,94]. Moreover, inflammatory levels have been associated with symptom severity in SCZ patients [95]. This profile is consistent with a pro-inflammatory environment that may promote the release of cfDNA [96] and therefore with the increase in cfDNA levels observed in SCZ patients. Importantly, as stated before, cfDNA can act as an endogenous DAMP stimulating inflammation through TLR9 and the stimulator of interferon genes (STING) pathway activation [97], thereby contributing to the persistent immune activation observed in SCZ [98]. In this context, cfDNA may reflect the cumulative cellular damage caused by oxidative DNA damage as evidenced by oxidation markers [91,99,100,101].

Interestingly, SCZ is characterized by mitochondrial abnormalities, such as reductions in mitochondrial gene expression, and alterations in size, number, and structure [91,102,103,104,105,106], that may contribute to mitochondrial damage and cf-mtDNA release. Notably, cf-mtDNA levels have been significantly correlated with changes in the Positive and Negative Syndrome Scale, associated with partial symptom improvement and reduced following antipsychotic treatment [92], suggesting their use as a tool to assist in the assessment of clinical progression in first-episode patients with SCZ. Finally, long-term psychotropic treatment was found to normalize mitochondria-related parameters in SCZ [107]. However, it is important to consider that antipsychotic exposure is associated with weight gain [108], insulin resistance [109], dyslipidaemia [110], and cardiovascular risk [111], all of which may independently influence cfDNA levels [10,112,113].

### 3.4. cfDNA as a Biomarker for Inflammation-Based Stratification in Psychiatric Disorders

Growing evidence across MDD, BD, and SCZ supports the notion that a subset of patients exhibits clinically relevant immune and inflammatory dysregulation [114,115]. This biological heterogeneity has limited the success of one-size-fits-all therapeutic approaches and has driven increasing interest in inflammation-based stratification strategies to better understand the role of inflammation in treatment response [4,116,117]. In this context, screening and stratification of psychiatric patients based on their inflammatory status may help guide personalized treatments [116].

cfDNA may be considered a particularly attractive biomarker, as it represents a signal of cellular damage following necrosis and apoptosis [118], immune activation [119], and stress [30,38], all conditions that may lead to systemic inflammation. Due to its possible role in activating and maintaining inflammation, cfDNA may serve as a biomarker for screening patients based on their inflammatory status. Moreover, targeted inhibition of cfDNA may represent an innovative approach to treat disorders characterized by an inflammatory status, such as psychiatric disorders, as it has already emerged as a promising therapeutic strategy for treating autoimmune diseases [120,121]. From a preventive perspective, cfDNA may also serve as a putative biomarker for population screening, enabling the identification of individuals with elevated inflammatory levels and, consequently, a higher risk of developing a psychiatric disorder. Additionally, elevated levels of inflammation in psychiatric conditions are often associated with increased medical comorbidity [122]. Within this context, cfDNA may provide integrative information from multiple biological systems [123] and disorders [10,57,124,125] and thus potentially capture cumulative allostatic load more effectively than traditional inflammatory markers alone, although this hypothesis has still not been validated in psychiatric cohorts.

Within inflammation-based stratification frameworks, cf-mtDNA may offer unique advantages over total cfDNA quantification. Indeed, cf-mtDNA represents a particularly powerful trigger of innate immune activation and may therefore help identify patients in whom mitochondrial damage and immune dysregulation are central drivers of psychopathology. Notably, mitochondrial abnormalities and dysfunction have been observed in various psychiatric disorders [6] and are reflected in cf-mtDNA levels. Moreover, cf-mtDNA levels increase in patients experiencing stress [31]. Thus, their detection may help identify the subgroup of patients characterized by higher stress levels, which are often poorly responsive to standard psychopharmacological treatments [126], but might benefit from inflammation-based intervention [127].

However, it is important to acknowledge that cfDNA should serve as a complementary measure, rather than a replacement, for already well-established inflammatory markers such as C-reactive protein and cytokine profiling. cfDNA could potentially contribute to an integrative approach capturing a broader dimension of biological stress and inflammation status not fully reflected by other inflammatory markers alone. This application, however, remains entirely at the theoretical stage in psychiatry and several important limitations must be acknowledged, such as reproducibility across laboratories, lack of standardized pre-analytical variables, and methodological heterogeneity.

## 4. Conclusions and Future Directions

To date, increasing evidence suggests that cfDNA may represent a promising common biomarker for cellular stress, immune activation, and metabolic dysregulation in several psychiatric disorders, including MDD, BD, and SCZ. Although current findings remain heterogeneous and sometimes contradictory, cfDNA appears to reflect core pathophysiological mechanisms such as oxidative stress, mitochondrial dysfunction, HPA-axis dysregulation, and systemic inflammation, which may help characterize biologically defined subgroups of patients. Its short half-life and dynamic responsiveness to psychological stress and treatment exposure further support its potential utility for short-term and longitudinal monitoring. Several methodological and biological factors may likely contribute to the literature heterogeneity, including small sample sizes, lack of standardized pre-analytical protocols, study design, insufficient control for metabolic and pharmacological confounders. More specifically, the sample type (plasma vs. serum) represents a critical pre-analytical variable that may lead to systematically different cfDNA concentrations, due also to lack of standardized pre-analytical protocols regarding sample preparation. Disease phase (acute vs. chronic) may also significantly influence cfDNA dynamics. Furthermore, medication use at the time of sampling should be more consistently controlled across studies, as specific drug classes could modulate cfDNA levels independently of the disease. Antipsychotics, mood stabilizers and antidepressants, could exacerbate metabolic dysregulations (including dyslipidaemia, insulin resistance and weight gain) [20] which may independently promote cfDNA release [10] and should therefore also be more accurately evaluated as potential confounders. At the same time, mood stabilizers such as lithium and valproate, as well as antidepressants, especially SSRIs and SNRIs, have been associated with immunomodulatory properties that may reduce cfDNA and cf-mtDNA release [75,83,84,85,128]. Finally, beyond age-related differences that could lead to increased cfDNA release, lifestyle variables such as smoke or physical activity, which can modulate cfDNA levels [19], should be accounted for.

While the dual role of cfDNA as both a product of cellular stress and an active driver of inflammatory signaling have been supported in other non-psychiatric conditions [13,14], direct evidence that demonstrate the same mechanism in psychiatric disorders is still limited. Future research should aim to integrate cfDNA into a multi-marker stratification framework, combining it with cytokine profiles, metabolic indices, multi-omics approaches, neuroimaging, and detailed clinical phenotyping. Studies focusing on cfDNA methylation may also offer insights into the tissue of origin, which may be helpful in determining whether immune, metabolic, or vascular compartments primarily drive inflammatory states in individual patients. Moreover, framing cfDNA as a biomarker for inflammation-based stratification should facilitate more targeted, personalized therapeutic approaches. Disentangling the involvement of cfDNA and cf-mtDNA in psychiatric disorders may enlighten their specific contribution to pathology development and the core mechanisms underlying each specific fraction. Standardization of sampling procedures and analytical pipelines will be essential to improve reproducibility. Finally, studies targeting cfDNA modulation may determine whether cfDNA could also act as a mediator and potential therapeutic target in the context of clinical improvement.

## Data Availability

No new data were created or analyzed in this study. Data sharing is not applicable to this article.

## Abbreviations

The following abbreviations are used in this manuscript:

cfDNA	Circulating cell-free DNA
DAMP	Damage-associated molecular pattern
Cf-mtDNA	Mitochondrial cell-free DNA
MDD	Major Depressive Disorder
BD	Bipolar Disorder
SCZ	Schizophrenia
HPA	Hypothalamic–pituitary–adrenal
TLR	Toll-like receptor
ROS	Reactive oxygen species
SNS	Sympathetic nervous system
PTSD	Post-traumatic stress disorder
STING	Stimulator of interferon genes
